# Tetrahydroxystilbene Glucoside Ameliorates Infrasound-Induced Central Nervous System (CNS) Injury by Improving Antioxidant and Anti-Inflammatory Capacity

**DOI:** 10.1155/2020/6576718

**Published:** 2020-01-06

**Authors:** Xuanxuan Zhou, Qian Yang, Fan Song, Linlin Bi, Jiani Yuan, Shaoyu Guan, Qi Yang, Siwang Wang

**Affiliations:** ^1^Department of Natural Medicine, School of Pharmacy, Air Force Medical University, Xi'an 710032, China; ^2^Collaborative Innovation Center for Chinese Medicine in Qinba Mountain, Xi'an 710038, China; ^3^Precision Pharmacy & Drug Development Center, Department of Pharmacy, The Second Affiliated Hospital of Air Force Medical University, Xi'an 710038, China; ^4^College of Life Science and Medicine, Northwest University, Xi'an 710069, China

## Abstract

**Background:**

Infrasound is a major threat to global health by causing injuries of the central nervous system (CNS). However, there remains no effective therapeutic agent for preventing infrasound-caused CNS injury. 2,3,5,4′-Tetrahydroxystilbene-2-O-*β*-D-glycoside (THSG) exerts protective function against CNS injuries and may have beneficial effects on infrasound-induced CNS impairment.

**Methods:**

A mouse model with CNS (oxidative stress-induced inflammation and neuronal apoptosis) injuries was established when the mouse was exposed to the infrasound of 16 Hz at 130 dB for 2 h each day and the duration of treatment was 8 d. The mice were divided into the control (CG, healthy mice), the model (MG, model mice), and the THSG (EG, experimental group, model mice treated with THSG) groups. The learning and memory impairments caused by infrasound were examined using a Morris water maze test. Lipid profiles, antioxidant biomarkers, and inflammatory cytokines in hippocampus tissue were measured by using corresponding ELISA kits. Meanwhile, BCL-2/BAX/caspase-3 signaling pathway was measured in the hippocampi and prefrontal cortex of the mouse brain using real-time qPCR and Western blot. Nissl's stain was used to measure neuronal necrosis in the hippocampi and prefrontal cortex of the mouse brain.

**Results:**

THSG significantly ameliorated the learning and memory impairments caused by infrasound. On the other hand, THSG improved lipid profiles, increased antioxidant properties by affecting the levels of superoxide dismutase (SOD), glutathione peroxidase (GSH-Px), catalase (CAT), and malondialdehyde (MDA), and displayed anti-inflammatory action via the downregulation of IL- (interleukin-) 6, IL-8, IL-10, TNF- (tumor necrosis factor-) *α*, and hs-CRP (high-sensitivity C-reactive protein) in the hippocampal tissues of the mouse model (*P* < 0.05). Additionally, Nissl's stain showed that THSG inhibited infrasound-induced neuronal necrosis in the hippocampi and prefrontal cortex. Besides, THSG exerted antiapoptosis function by upregulating the level of Bcl-2 and downregulating the levels of BAX and caspase-3 in the hippocampi.

**Conclusion:**

THSG may be an effective anti-infrasound drug against CNS injury by improving antioxidant, anti-inflammatory, antiapoptosis, and antinecrosis capacities. Further research is still needed to confirm the exact molecular mechanism.

## 1. Introduction

**Figure 1:**
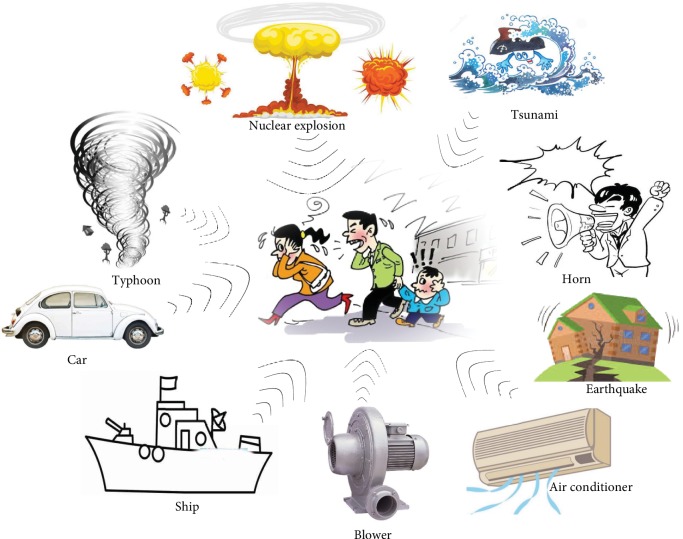
Categories of infrasound events.

**Figure 2:**
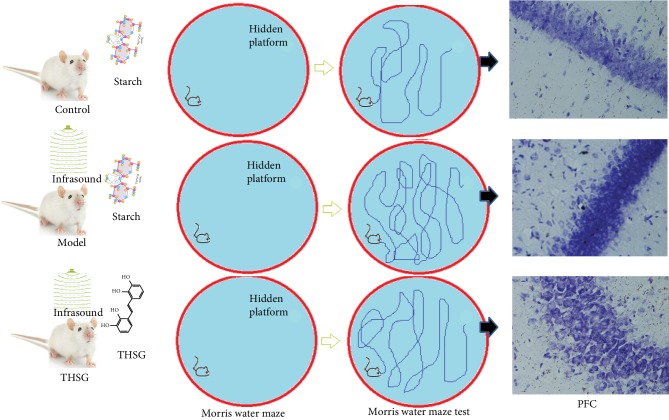
Schematic diagram of Morris water maze test among different groups. *n* = 8 for each group.

Infrasound is the sound with the frequencies lower than 20 Hz [1] and can be produced by ocean waves, earthquakes, wind, apparatuses, home utilizations, buses, cars, and so on (Figure 1), [2]. With the quick development of industry, transportation vehicles, and electrical instruments, infrasound-induced injuries have increased significantly and are not only important noise pollution anymore [3]. Wind turbine low-frequency noise is a kind of infrasound that can produce community harmful to human health. In the field measurements, spectral peaks could be detected for 10 km away from wind turbines [4]. Infrasound was responsible for the onset of adverse health effects self-reported by some persons who were near with wind turbines [2]. Some relationships were found between exposure to infrasound and annoyance, sleep-related problems, concentration difficulties, and headache in the population who lived in a range of infrasound [5]. Infrasound pollution can cause human organ dysfunction and cardiac injury [6]. Animal experiments showed that infrasound destroyed hearing, cardiac, respirational, gastrointestinal, and circulation systems [7]. Particularly, infrasound impaired the central nervous system (CNS) and cognitive abilities [8].

However, it is difficult to prevent human beings from infrasound pollution because it cannot be detectable in most cases [9, 10]. Thus, it is necessary to explore novel drugs that effectively protect CNS against infrasound-caused injury pollution. 2,3,5,4′-Tetrahydroxystilbene-2-O-*β*-D-glucoside (THSG) is a bioactive ingredient from a traditional Chinese herbal medicine *Polygonum multiflorum Thunb* (PMT). THSG has been reported to be beneficial for human health with a great number of pharmacological properties including antioxidant [11], anti-inflammation [11], free radical scavenging, and cardioprotective effects [5, 12, 13]. THSG have shown its potential for protective effects against *β*-amyloid deposition and memory deficits in a dementia mouse model [14]; THSG reduced cognitive impairment and inhibited the overexpression of hippocampal amyloid precursor protein (APP) [15]. THSG improved the learning and memorizing ability of aged mice and reduced senile plaque deposition induced by reducing APP [16]. Recently, we found that THSG provided protective effects by upregulating the expression of SOD and scavenging reactive oxygen species (ROS) as a potential antioxidant [17] while ROS may be associated with infrasound-induced brain injury. Infrasound-caused CNS injury was related to free radical accumulation and a high level of peroxidation in the brain cortex [18]. Infrasound exposure destroyed oxidation-antioxidant balance and increased lipofuscin accumulation in neurons of the mouse brain cortex [19]. Oxidative stress can induce apoptosis [20], and the apoptosis may result in the loss of postsynaptic proteins [21]. Postsynaptic current was modulated by postsynaptic proteins [22] while postsynaptic neurons modulate learning [23] and their dysfunction will impair memory [24]. Thus, oxidative stress can impair learning and memory by affecting postsynaptic activity.

Infrasound can affect the liver and cause the increase of lipid peroxidation [25, 26], which results in the changes of total cholesterol (TC), triglyceride (TG), low-density lipoprotein cholesterol (LDL-C), and high-density lipoprotein cholesterol (HDL-C) [27, 28]. Further work also showed that infrasound exposure increased lipid peroxidation in the kidneys [29]. On the other hand, oxidative stress is often associated with inflammatory responses [30, 31]. The interplay of oxidative and inflammation may play an important role in CNS injury.

However, little information is available in the literature regarding the mechanism of nervous protective effects for THSG against infrasound-induced CNS injury. Therefore, we performed experiments to explore the protective effects of THSG against infrasound-induced CNS injury by exploring its antioxidant and anti-inflammatory properties.

## 2. Materials and Methods

### 2.1. Materials

All chemical reagents with standard biochemical quality including THSG were purchased from Sigma (MO, USA).

### 2.2. Establishment of a Mouse Model with Infrasound-Induced CNS Injury

The experiment was approved by Animal Research Ethics Committee of the Fourth Military Medical University (Approval No. JLU24589XYZ). c57BL/6J male mice (18-20 g) were obtained from the Animal Center of the Fourth Military Medical University (Xi'an, China). The mice were given ad libitum access to food in an animal room (12/12 h light/dark cycle at 22 ± 1°C). Infrasound instrument (Infrasonic Qigong Machine (QGM) 4.0; China Healthways Institute, San Clemente, CA, USA) was comprised of an infrasound producer and an infrasound detector. The instrument produced the infrasound with frequencies less than 20 Hz. The mice were evenly assigned into control (CG), infrasound-induced model (MG), and THSG (EG, infrasound induction +100 mg/kg THSG) groups, and *n* = 8 in each group. THSG concentration was chosen from a previous report [32] and low concentration was used. Infrasound model was established when the mice were exposed to the infrasound of 16 Hz at 130 dB for 2 h each day and the whole treatment was 8 d.

### 2.3. THSG Administration

THSG was solubilized in 0.85% NaCl solution and was administered to mice in the THSG group via intragastric administration. This experiment process was repeated for 7 d (intragastric administration was conducted for 24 h before infrasound exposure on the last day.).

### 2.4. Spatial Learning and Memory Test

All mice received the Morris water maze (MWM) test after the establishment of infrasound-induced CNS injury model [33]. An escaping platform was placed in the quadrant IV of a pool at one cm under water surface on from 1 to 4 d and removed on 5 d. If a mouse could not find the platform within one min, it would be placed on the platform for 15 s. The mice were trained for 4 d to find the plate. All mice were given 1 min to find the place where the platform was previously placed. The spatial learning scores (latency and path length) were documented.

### 2.5. Measurement of Lipid Profiles

After the MWM test, the mice were killed by decapitation, and the left and right hippocampi were isolated from the brain hemispheres immediately. Eight mg hippocampi were homogenized in 0.85% saline solution, lysed by using glass beads lysis (425–600 *μ*m; Sigma Cat No. G-8772), and then centrifuged at 13,000 × *g* for 10 min at 4°C. Forty *μ*L supernatants were collected to estimate the lipid profile. Mouse total cholesterol (TC) ELISA Kit (Cat. No. MBS269999), mouse triglycerides (Triglycerides, TG) ELISA Kit (Cat. No. #MBS1601281), mouse high-density lipoprotein cholesterol (HDL-C) ELISA Kit (Cat. No. #MBS268119), and mouse low-density lipoprotein cholesterol (LDL-C) ELISA Kit (Cat. No. #MBS748297) were purchased from MyBioSource, Inc. (San Diego, CA, USA). The changes of lipid profiles were explored by measuring TC, TG, HDL-C, and LDL-C in hippocampus tissues on an automatic analyzer (Olympus Automated Chemistry Analyzer AU2700, Tokyo, Japan).

### 2.6. Measurement of Antioxidant Capacities

Above 30 *μ*L supernatants were collected to estimate the levels of catalase (CAT), malondialdehyde (MDA), malondialdehyde (MDA), superoxide dismutase (SOD), and glutathione peroxidase (GSH-Px). The levels of CAT (CAT Assay Kit, R&D Systems Ltd., MI, USA), MDA (malondialdehyde assay kit, Beyotime Institute of Biotechnology, Beijing, China), SOD (SOD determination kit, Fluka, St. Louis, MO, USA), and GSH-Px (GSH-Px assay kit, Nanjing Jiancheng Biology Research Institute, Nanjing, China) were measured by using corresponding kits.

### 2.7. Measurement of Inflammatory Cytokines

Above 30 *μ*L supernatants were collected to measure the levels of inflammatory cytokines. ELISA kits for interleukin- (IL-) 6 (Cat. No. ab46100), IL-8 (Cat. No. ab46032), IL-10 (Cat. No. ab108870), and tumor necrosis factor- (TNF-) *α* (Cat. No. ab208348) were purchased from Abcam (Cambridge, MA, USA) and measured at 450 nm in a microplate reader (BioTek, Bad Friedrichshall, Germany). The high-sensitivity C-reactive protein (hs-CRP) was determined by immunoturbidimetry, and the test reagent was a high-sensitivity C-reactive protein reagent from Deling Company (Tangshan, China).

### 2.8. Nissl's Staining

Nissl's staining can show cellular necrosis [34] but not apoptosis. The increase in the number of Nissl bodies (chromatin granules) showed the reduction in neuronal necrosis [35]. Nissl staining demonstrated that the number of surviving neurons and Nissl bodies was lacking in the necrosis status of neurons [36]. Two mg hippocampi or 10 mg prefrontal cortex (PFC) from each mouse was fixed in 10% formalin at 4°C for 10 h and cut into 300 *μ*m thick slices (*n* = 8 for each group). For histological analysis with Nissl's staining, the hippocampus or PFC slices were deparaffinized in xylene and hydrated through a series of alcohol, rinsed in distilled water, and were incubated with 0.1% cresyl violet solution for 10 min. The neurons in the PFC and hippocampi were counted under a light microscope using 100x and 400x magnifications. The cells with round shape, Nissl's staining in cytoplasm, loose chromatin, and prominent nucleoli were considered as normal neurons; the cells with shrunken shape, condensed, or without Nissl's staining were considered as injured neurons.

### 2.9. RT-PCR Analysis of Apoptosis-Related Genes in the Hippocampi

Total RNA of above 50 *μ*L supernatants was extracted from by using a RNA extraction kit (TaKaRa Biotechnology (Dalian) Co., Ltd., Dalian, China). Specific primers for *Bcl-2*, *bax*, and *caspase-3* genes were designed and synthesized by TaKaRa (*β-actin*, forward primer: CACGATGGAGGGGCCGGACTCATC, reverse primer: TAAAGACCTCTATGCCAACACAGT; *Bcl-2*, forward primer: GGTGAACTGGGGGAGGATTG, reverse primer: GCATGCTGGGGCCATATAGT; *bax*, F: GGCGATGAACTGGACAACAA, R: CAAAGTAGAAAAGGGCAACC; and *Caspase-3*, forward primer: GGACCTGTGGACCTGAAAAA, reverse primer: GCATGCCATATCATCGTCAG). Reverse transcription was performed as follows: 42°C, 1 h; 95°C, 5 min. cDNA was used for a multiplex qRT-PCR by using real-time PCR system instrument (Thermo Fisher Scientific, Waltham, MA, USA) and SYBR Green Master Mix (Applied Biosystems, Foster City, CA, USA). RT-PCR reactions were performed under the following conditions: one cycle of 95°C for 5 min, followed by 45 cycles of 95°C for 10 s, 60°C for 15 s, and 72°C for 20 s. *β*-Actin was used as a control. Fold change was calculated as 2^-*ΔΔ*Ct^.

### 2.10. Western Blot Analysis

Western blot was performed to measure relative protein levels of BAX, Bcl-2, and caspase-3 by using above 50 *μ*L supernatants. The protein solution was separated using SDS-PAGE and transferred to a PVDF membrane (Millipore, Shanghai, China), which was blocked for 2 h using a 5% skim milk. The membrane was incubated with the antibodies caspase-3 (1 : 1000; Cat. No. #9661), BAX (1 : 1000; Cat. No. #2870), and Bcl-2 (1 : 1000; Cat. No. #2772, Cell Signaling Technology, Boston, MA, USA) at 4°C for 24 h. The membranes were further incubated with a secondary antibody HRP goat-anti rabbit IgG (Thermo Scientific, Waltham, MA, USA, Cat. No 31460). Protein bands detected enhanced chemiluminescence (Millipore, Shanghai, China). The relative protein levels were calculated by using *β*-actin as a control. Caspase positive immunostaining was calculated by using the cut-off of 300 AU. The intensity of protein bands was examined using ImageJ software (version 2.0, NIH, MD, USA).

### 2.11. Statistical Analysis

All data were presented as the mean values ± standard deviation (S.D.) and analyzed by using the software (SPSS v20.0, Stanford, CA, USA). The eEPSC current data were analyzed using Clampfit 10.3, and the average of the 10 continuous recordings was calculated as the final current amplitudes. The frequency and magnitude of mEPSC were analyzed using a Mini Analysis Program (Synaptosoft, Leonia, NJ, USA). One-way ANOVA and LSD post hoc test were used to determine the statistical difference with *P* < 0.05.

## 3. Results

### 3.1. THSG Improved Spatial Learning and Memory Abilities

**Figure 3:**
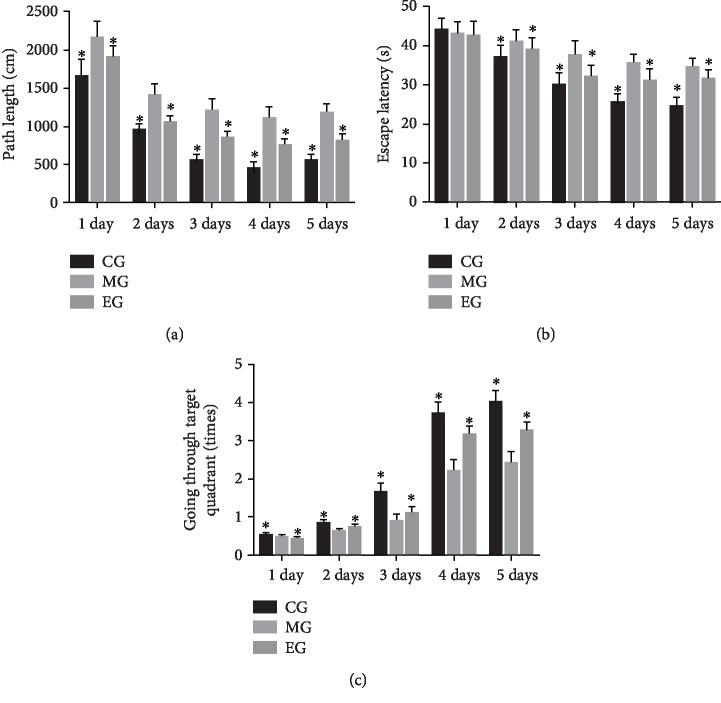
The effects of THSG on spatial learning and memory. (a) Path length (cm). (b) Escape latency (s). (c) The times of going through the target quadrant. *n* = 8 for each group and ^∗^*P* < 0.05 vs. the model group.

Figure 2 schematically shows that an MWM test was used among different groups, and their pathway length for finding the platform in the pool and neural necrosis was measured. Compared to the control group, there was a significant increase in the path length in the model group, whereas THSG reduced the length (Figure 3(a), *P* < 0.05). The mice in the control group had shorter escape latency than in the model group. THSG treatment reduced the latency when compared with the model group (Figure 3(b), *P* < 0.05). In contrast, the mice in the control group had more times for going through target quadrant than in the model group. THSG treatment increased the times when compared with the model group (Figure 3(c), *P* < 0.05). The results suggested that the infrasound impaired memory ability and THSG treatment improved the symptoms.

### 3.2. THSG Treatment Improved Lipid Profiles

The changes of lipid profiles in mouse hippocampi after infrasound exposure were investigated. The results showed that infrasound exposure caused a dramatic decrease in the level of HDL-C in hippocampi and increased the level of TG, TC, and LDL-C in hippocampi when compared to the normal control (Table 1, *P* < 0.05). THSG increased the brain levels of HDL-C and reduced the levels of TG, TC, and LDL-C when compared with the mouse model with infrasound-induced CNS injury (Table 1, *P* < 0.05). The results suggest that THSG treatment improved the lipid profiles of the moue model with CNS injury.

### 3.3. THSG Treatment Increased Antioxidant Capacities

The changes in the activity of antioxidant and oxidative biomarkers in mouse hippocampi after infrasound exposure were investigated. Higher activity of SOD, GSH-Px, and CAT is closely associated with antioxidant defense [37]. The results showed that infrasound treatment caused a dramatic decrease in the activities of SOD, GSH-Px, and CAT in hippocampal tissues, and increased the level of malondialdehyde (MDA) in hippocampal tissues when compared to the control group (*P* < 0.05). THSG increased the levels of SOD, GSH-Px, and CAT and reduced the level of MDA when compared with the CNS injury model (Table 2, *P* < 0.05). The results suggest that THSG treatment increased antioxidant capacities of model mice.

### 3.4. THSG Treatment Increased Anti-Inflammatory Capacities

The changes in the levels of cytokines in mouse hippocampi after infrasound exposure were investigated. Before the model establishment and treatment, the levels of all inflammatory cytokines were similar among all groups (Table 3, *P* > 0.05). After the model establishment, the results showed that infrasound treatment caused a dramatic increase in IL-6, IL-8, IL-10, TNF-*α*, and hs-CRP when compared to the control group (Table 3, *P* < 0.05). THSG treatment reduced the levels of IL-6, IL-8, IL-10, TNF-*α*, and hs-CRP in hippocampal tissues when compared with the CNS injury model (Table 3, *P* < 0.05). The results suggest that THSG treatment increased anti-inflammatory capacities of the hippocampi of the model mice.

### 3.5. THSG Inhibited Infrasound-Induced Neuron Necrosis of PFC Region in the Mouse Model

**Figure 4:**
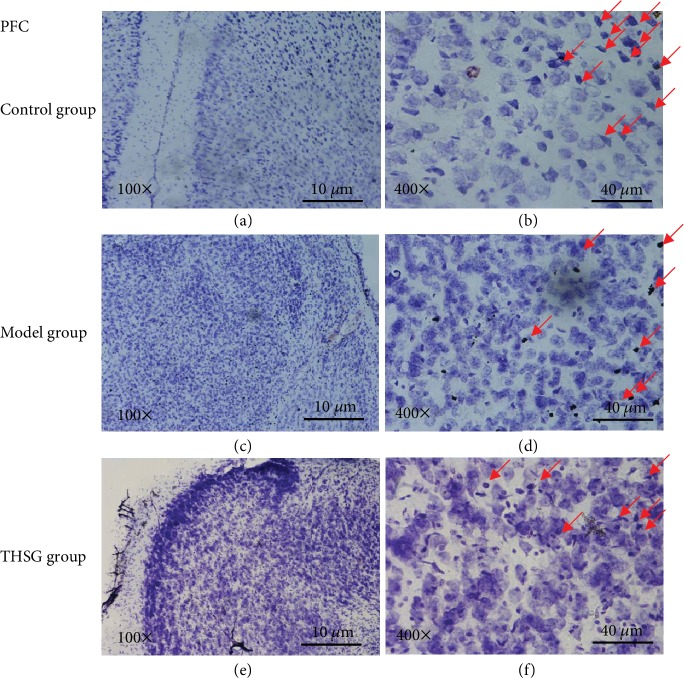
The effects of THSG on infrasound-induced neuron apoptosis of prefrontal cortices (PFC) region in the mouse model. The results were analyzed from three aspects. Quantity: the control (a, b) and THSG (e, f) groups had more neurons than the model group (c, d). Shape: the shapes of cells in the control group (a, b) were rounder than the model group (c, d), and the cells in the THSG group (e, f) also had a more perfect shape than the model group (c, d). Definition: big cells, staining cytoplasm, loose chromatin, and prominent nucleoli were showed in the control group (a, b) while small cells and condensed or even no staining cytoplasts were observed in the model group while the situation became better in the THSG group. Nissl bodies (chromatin granules) stand for the activity of protein synthesis and showed in red arrows and are lacking in necrotic cells. *n* = 8 for each group.

Neuronal necrosis in mouse PFC was measured using Nissl's staining. In the control group, there were many large Nissl bodies. Compared with controls (Figures 4(a) and 4(b)), infrasound treatment induced necrotic neurons with less Nissl bodies in PFC region of the mouse model (Figures 4(c) and 4(d)). THSG pretreatments at 100 mg/kg THSG significantly increased the number of Nissl bodies (Figures 4(e) and 4(f)).

### 3.6. THSG Inhibited Infrasound-Induced Neuron Necrosis of Hippocampus Region in the Mouse Model

**Figure 5:**
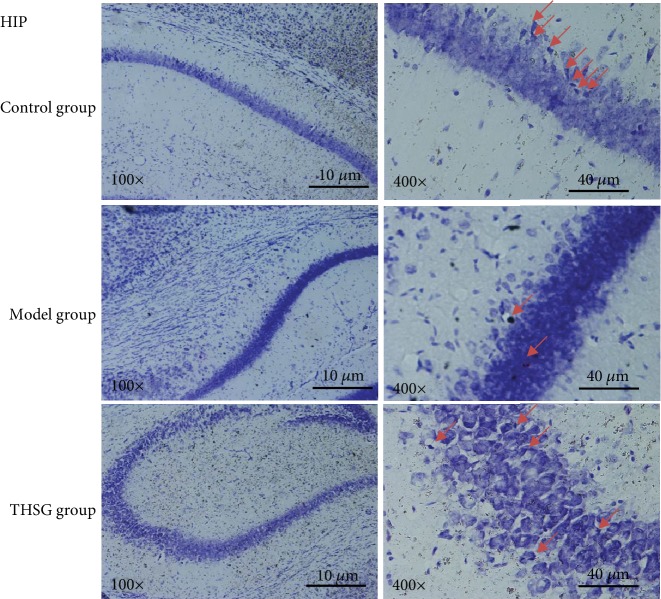
The effects of THSG on infrasound-induced neuron apoptosis of hippocampus (HIP) region in the mouse model. The results were analyzed from three aspects. Quantity: the control (a, b) and THSG (e, f) groups had more neurons than the model group (c, d). Shape: the shapes of cells in the control group (a, b) were rounder than the model group (c, d), and the cells in the THSG group (e, f) also had a more perfect shape than the model group (c, d). Definition: big cells, staining cytoplasm, loose chromatin, and prominent nucleoli were showed in the control group (a, b) while small cells and condensed or even no staining cytoplasts were observed in the model group while the situation became better in the THSG group. Nissl bodies (chromatin granules) stand for the activity of protein synthesis and showed in red arrows and are lacking in necrotic cells. *n* = 8 for each group.

Nissl's staining showed that infrasound caused neuronal necrosis in mouse hippocampi. When compared with controls (Figures 5(a) and 5(b)), infrasound treatment increased the amounts of necrotic neurons compared with the control in hippocampus region (Figures 5(c) and 5(d)). THSG pretreatments (100 mg/kg) significantly increased the number of Nissl bodies (Figures 5(e) and 5(f)).

### 3.7. The Effects of THSG on Relative mRNA Levels of Apoptosis-Related Genes

**Figure 6:**
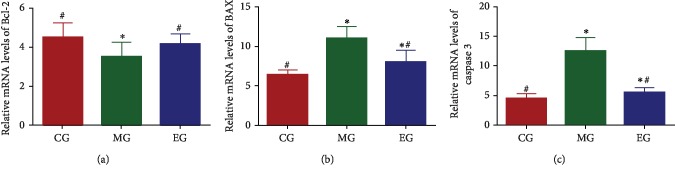
The effects of THSG on relative mRNA levels of Bcl-2/BAX/caspase-3. (a) The effects of THSG on relative mRNA levels of Bcl-2. (b) The effects of THSG on relative mRNA levels of BAX. (c) The effects of THSG on relative mRNA levels of caspase-3. *n* = 8 for each group. ^∗^*P* < 0.05 vs. the control group and ^#^*P* < 0.05 vs. the model group.

Bcl-2 [38], BAX [39], and caspase-3 [40, 41] have been reported to be associated with oxidative stress and apoptosis. Infrasound treatment reduced relative mRNA level of *Bcl-2* and a sharp increase in relative mRNA levels of *bax* and *caspase-3*. Compared with the MG group, relative mRNA level of *Bcl-2* was increased and the levels of *bax* and *caspase-3* were reduced in the 100 mg/kg EG group (Figure 6).

### 3.8. The Effects of THSG on Bcl-2/BAX/Caspase-3 Signaling Pathway

**Figure 7:**
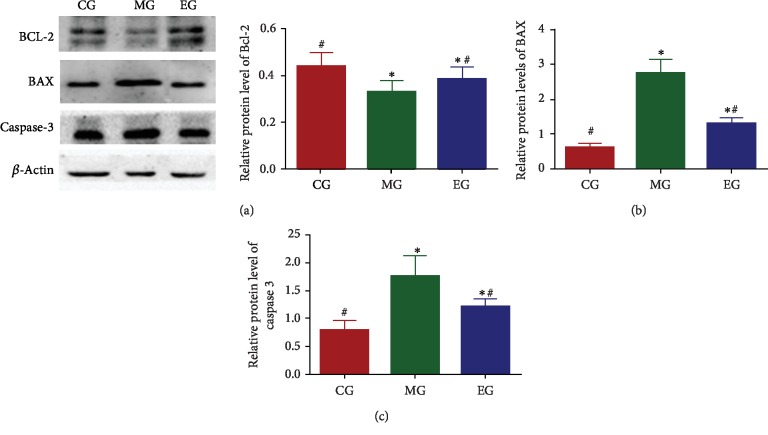
The effects of THSG on relative protein levels of Bcl-2/BAX/caspase-3. (a) The effects of THSG on relative protein levels of Bcl-2. (b) The effects of THSG on relative protein levels of BAX. (c) The effects of THSG on relative protein levels of caspase-3. *n* = 8 for each group. ^∗^*P* < 0.05 vs. the control group and ^#^*P* < 0.05 vs. the model group.

Infrasound treatment reduced Bcl-2 level and increased the levels of BAX and caspase. Comparatively, THSG (100 mg/kg) increased the level of Bcl-2 and reduced the levels of BAX and caspase-3 (Figure 7). THSG showed antiapoptosis activity in infrasound-induced CNS injury model by affecting the levels of Bcl-2/BAX/caspase-3.

## 4. Discussion

Infrasound is widely existed in our environment and cannot be detected directly but it can induce brain injury by increasing oxidative stress [18] and cell apoptosis in hippocampi [42, 43]. The disorder of CNS is closely associated with infrasound effect [44]. Infrasound impaired animal learning and memory abilities [45], inhibited adult neurogenesis in hippocampi, and induced neuronal injury [46]. Cognitive ability was affected by the infrasound of 16 Hz at 130 dB [8]. Hippocampi and PFC play an important role in behavioral and cognitive psychology [47, 48]. The present finding also demonstrated that infrasound treatment increased the oxidative stress via the upregulation of MDA and downregulation SOD, GSH-Px, and CAT (Table 2) and increased cell apoptosis via the upregulation of BAX and caspase-3 and downregulation of Bcl-2 (Figures 6 and 7) in hippocampi and cellular necrosis in hippocampi and FPC cells. THSG treatment improved the status of cellular oxidative stress, apoptosis, and necrosis caused by infrasound.

What is the effect of THSG on animal autonomic behavior and nerve excitation? The exact mechanism remains unclear. Mammalian brain is susceptible to free radicals [49] and ROS oxidize and disrupt brain homeostasis via the induction of cell death. Infrasound also can cause brain injury by accumulating ROS [18]. Most ROS, such as OH, H_2_O_2_, and O_2_^−^, can be produced by infrasound [50]. Lipid accumulation is associated with ROS development [51], induces the production of mitochondrial ROS according to a previous report [52], and may increase oxidative stress in the cell. THSG therapy reduced lipid accumulation and showed potential protective agent for infrasound-induced injury as a novel ROS scavenger, a phytoestrogen. We hypothesized that the protective effect of THSG against infrasound-induced injury was their ability to counteract ROS, such as hydroxyl radical (OH^·^) and superoxide radical (O_2_^·-^), and other peroxyl radicals, and limited the oxidative injury to mouse hippocampal tissues. This process occurred because THSG slowed the accumulation of intracellular ROS, counteracted the overexpression of inducible nitric oxide synthase as well as neuronal nitric oxide syntheses [53].

Memory deficiency can be caused by inflammation [54]. Inflammation can be caused by oxidative stress, and the interplay of oxidative stress and inflammation may play an important role in the disease. Thus, THSG may have protective function for memory deficiency by improving the spatial learning and memory performance via the upregulation of anti-inflammatory properties (Table 3). Furthermore, the levels of inflammatory cytokine were increased from the CG group to the EG group and reached the highest level in the MG group (Table 3). These results suggest the potential anti-inflammatory role of THSG in the prevention of memory deficiency.

Infrasound induced neuronal apoptosis, which should be associated with memory deficiency [55]. One hundred mg/kg THSG treatment inhibited neuronal apoptosis in mouse hippocampi (Figures 5 and 4). Bcl-2 is an important antiapoptotic factor [56], and BAX and caspase-3 are common proapoptotic factors [57, 58]. The present findings showed THSG treatment upregulated the Bcl-2 level and downregulated the level of BAX and caspase-3 (Figures 6 and 7). According to previous reports and the present findings, infrasound may increase oxidative stress and cause apoptosis in the hippocampal tissues of the animal model [59, 60].

There were some limitations of the present work. The present work is still limited with animal model experiments, and the effects of THSG on clinical trial of infrasound-induced CNS injury remain unclear. THSG showed antioxidant, anti-inflammatory, antiapoptosis, and antinecrosis properties, which may be associated with specific antioxidant and anti-inflammatory signaling pathway. Thus, the exact molecular mechanism will be explored in the future work.

## 5. Conclusions

The results showed that THSG inhibited infrasound injury by reducing the path length along with escape latency and increasing the frequency of going through the target quadrant. Meanwhile, THSG improved antioxidant and anti-inflammatory capacities in hippocampal tissue of the mouse models and reduced infrasound-caused neuronal apoptosis and necrosis in the hippocampi and or PFC regions. THSG may be developed a potential drug against the infrasound injury on the CNS system, but further research is still needed in the future.

## Figures and Tables

**Table 1:** Effects of THSG on levels of TC, TG, HDL-C, and LDL-C in the hippocampus of the mice.

Groups	TC/(mmol/L)	TG/(mmol/L)	HDL-C/(mmol/L)	LDL-C/(mmol/L)
Before the model establishment and treatment				
CG	2.40 ± 0.16	1.22 ± 0.11	1.42 ± 0.12	1.52 ± 0.16
EG	2.43 ± 0.14	1.20 ± 0.12	1.39 ± 0.15	1.58 ± 0.15
MG	2.38 ± 0.17	1.19 ± 0.13	1.41 ± 0.16	1.55 ± 0.17
After the model establishment and treatment				
CG	2.46 ± 0.22^c^	1.23 ± 0.15^c^	1.49 ± 0.18^c^	1.69 ± 0.22^c^
EG	2.53 ± 0.56^c^	1.27 ± 0.18^c^	1.56 ± 0.19^c^	1.87 ± 0.49^c^
MG	4.2 ± 0.60^a,b^	1.55 ± 0.17^a,b^	1.10 ± 0.25^a,b^	2.58 ± 0.29^a,b^

Note: *n* = 8 for each group. ^a^*P* < 0.05 vs. the CG group, ^b^*P* < 0.05 vs. the EG group, and ^c^*P* < 0.05 vs. the MG group.

**Table 2:** Effects of THSG on antioxidant capacity in the hippocampal tissues of the mice.

Groups	MDA/(nmol/mg)	SOD/(U/L)	GSH-Px/(U/mg)	CAT/(U/g)
Before the model establishment and treatment				
CG	5.64 ± 0.52	97.62 ± 8.23	224.29 ± 30.17	50.23 ± 2.01
EG	5.58 ± 0.46	92.53 ± 8.12	226.43 ± 28.62	48.17 ± 1.86
MG	5.60 ± 0.49	94.68 ± 7.87	227.55 ± 29.32	52.02 ± 2.25
After the model establishment and treatment				
CG	5.76 ± 0.66^b,c^	110.30 ± 8.34^b,c^	288.5 ± 43.76^b,c^	55.87 ± 2.05^b,c^
EG	7.29 ± 1.07^a,c^	99.69 ± 8.61^a,c^	238.28 ± 36.88^a,c^	45.69 ± 10.91^a,c^
MG	8.94 ± 2.33^a,b^	77.97 ± 6.19^a,b^	173.12 ± 23.51^a,b^	33.32 ± 11.97^a,b^

Note: *n* = 8 for each group. ^a^*P* < 0.05 vs. the CG group, ^b^*P* < 0.05 vs. the EG group, and ^c^*P* < 0.05 vs. the MG group.

**Table 3:** Effects of THSG on inflammatory cytokines in the hippocampal tissues of the mice.

Groups	IL-6 (pg/mL)	IL-8 (ng/mL)	IL-10 (pg/mL)	TNF-*α* (ng/mL)	Hs-CRP (*μ*g/mL)
Before the model establishment and treatment					
CG	98.76 ± 11.37	0.38 ± 0.18	222.91 ± 85.21	2.44 ± 1.08	3.72 ± 1.34
EG	99.08 ± 10.24	0.35 ± 0.14	228.65 ± 78.49	2.48 ± 1.12	3.86 ± 1.26
MG	99.29 ± 10.29	0.39 ± 0.18	230.25 ± 81.21	2.46 ± 1.10	3.79 ± 1.03
After the model establishment and treatment					
CG	100.54 ± 12.18^b,c^	0.36 ± 0.20^b,c^	224.82 ± 88.48^b,c^	2.52 ± 1.31^b,c^	3.77 ± 1.39^b,c^
EG	108.85 ± 12.23^a,c^	0.54 ± 0.19^a,c^	283.50 ± 76.39^a,c^	3.47 ± 1.43^a,c^	5.06 ± 1.26^a,c^
MG	119.10 ± 14.33^a,b^	0.62 ± 0.23^a,b^	309.61 ± 79.26^a,b^	3.83 ± 1.58^a,b^	5.65 ± 1.53^a,b^

Note:*n* = 8 for each group. ^a^*P* < 0.05 vs. the CG group, ^b^*P* < 0.05 vs. the EG group, and ^c^*P* < 0.05 vs. the MG group.

## Data Availability

All data can be available on the inquiry for the corresponding authors.
